# Supplementary material for: Machine learning predicts lifespan and suggests underlying causes of death in aging C. elegans
Source: Commun Biol. 2025 Nov 21;8:1630. doi: 10.1038/s42003-025-09012-9 (PMC12638908; doi:10.1038/s42003-025-09012-9)
Supplement: Supplementary file 2 — Supplementary Information [file 42003_2025_9012_MOESM2_ESM.pdf]

Supplementary Information

Machine learning predicts lifespan and suggests underlying causes of death in aging *C. elegans*

Contents Summary

Supplementary Results

Supplementary Figure 1. Quantification of age-related pathologies in aging *C. elegans.*

Supplementary Figure 2. Effects on senescent pathologies of axenic culture (putative DR regimen).

Supplementary Figure 3. Mutation of *daf-2* differentially suppresses senescent pathology.

Supplementary Figure 4. Effects on pathology development of tissue-specific *daf-16* rescue and *hsf-1* inhibition.

Supplementary Figure 5. Effects of tissue-specific rescue of *daf-16* on senescent pathology in *daf-16; daf-2(e1370)* mutants.

Supplementary Figure 6. Evidence that protein synthesis and mitochondrial function promote pathology development.

Supplementary Figure 7. Ribosomal protein S6 kinase has small effects on pathology development.

Supplementary Figure 8. Senescent pathology can be used to predict lifespan.

Supplementary Figure 9. Predictive power of the ML model.

Supplementary Table 1. Pathology scoring system.

Supplementary Table 2. All lifespan data sources and pathology data sources collated and used in ML analysis.

Other supplementary files

Supplementary Dataset 1: Raw pathology and lifespan data original to this study.

Supplementary Dataset 2: Raw pathology and lifespan data used to build the ML model.

Supplementary results

Effects of axenic culture on senescent pathology

We examined how interventions that alter lifespan affect the rate of development of senescent pathology. Dietary restriction (DR) increases lifespan and delays senescent pathology in a number of organisms[1]. Several putative *C. elegans* DR regimens have been described[2]. One such is culture on nutrient-rich, semi-defined axenic medium (i.e. lacking a microbial food source), which causes severe retardation of development and large increases in lifespan and therefore resembles a DR effect[3,4]. We employed a solid medium protocol where larvae are raised on UV-killed *E. coli* OP50 to support normal growth and then transferred onto axenic medium plates (no *E. coli*) at adulthood[5]. This resulted in almost complete suppression of pharyngeal deterioration, PLPs and intestinal atrophy, and partial suppression of uterine tumors (Supplementary Fig. 2a-d). Gonad atrophy was not measured as axenic culture inhibited gonadal development.

Selective effects on pathology of different *daf-2* alleles

Reduction of insulin/IGF-1 signaling (IIS), as in *daf-2* insulin/IGF-1 receptor mutants, greatly increases *C. elegans* lifespan, and this effect is dependent upon the DAF-16 FOXO transcription factor[6]. Mutation of *daf-2* suppresses development of diverse pathologies, including gonadal and pharyngeal deterioration[7], late-life bacterial infection in the pharynx[8,9], intestinal atrophy and yolk accumulation[10,11]. Inhibiting bacterial infection or yolk synthesis is sufficient to extend lifespan[12–14], suggesting that reduced IIS increases lifespan at least partly by blocking development of pathology.

We previously compared effects on intestinal atrophy and yolk pool accumulation of two *daf-2* mutations, a less pleiotropic (class 1) allele *daf-2(e1368)*, and a more pleiotropic (class 2) allele *daf-2(e1370)* (Ref 15). This showed that *e1368* modestly delayed both pathologies, but *e1370* strongly suppressed them[11]. Here we extend this analysis to the remaining three pathologies. Notably, the five pathologies were not regulated by IIS as a group, in tandem with one another. For example, in *e1370* mutants, development of pharyngeal and gonadal pathology was delayed but eventually reached maximal wild-type levels, whereas for intestinal atrophy and yolk pools they did not. Moreover, little reduction in uterine tumor growth was detected in either mutant (Supplementary Fig. 3a-e). The lack of effect was also somewhat unexpected, given the previous observation that *daf-2* also inhibits formation of chromatin masses within uterine tumors[16]; it suggests that *daf-2* reduces chromatin mass formation without reducing tumor size.

All suppression of pathology by *daf-2(e1370)* was fully dependent upon *daf-16* FOXO, as is the case for *daf-2* longevity[17], consistent with the possibility that IIS shortens lifespan at least in part by causing senescent pathology (Supplementary Fig. 3f-j).

These findings imply that although wild-type IIS promotes all five senescent pathologies, it does not do so equally. Rather, IIS strongly promotes intestinal atrophy and yolk accumulation, modestly promotes gonadal atrophy and pharyngeal deterioration, and has little effect on uterine tumor growth. Also, different alleles differ in terms of severity of

suppression of pathology as well as mortality: *e1370* has stronger effects than *e1368* on both pathology (including pharyngeal infection, gut atrophy and PLPs) and lifespan[8,9,11].

Next we investigated the role of the *daf-16* FOXO transcription factor gene in the development of senescent pathology. Mutation of *daf-16* slightly shortens lifespan[18] and fully suppresses *daf-2* longevity[17]. The null mutation *daf-16(mgDf50)* reduced pharyngeal deterioration slightly and did not significantly affect gonad atrophy or tumor formation, but did aggravate PLPs and intestinal atrophy (Supplementary Fig. 4a-e). This is in agreement with an earlier study showing little effect of *daf-16* on the pharynx and germline[7]; and our observation that *daf-16(0)* accelerates PLPs and intestinal aging[11].

We also examined the effects of knockdown of the *hsf-1* heat shock factor, another transcription factor that mediates the effect of *daf-2* on lifespan[19]. It was previously shown that *hsf-1* RNAi substantially shortens lifespan and accelerates pharyngeal pathology[7]. Although we could not confirm the effects of *hsf-1* RNAi on pharyngeal pathology, we did observe accelerated gonad atrophy and intestinal atrophy (Supplementary Fig. 4f-j). These results could imply that loss of *daf-16* or *hsf-1* shortens lifespan by promoting pathology development, and support the earlier deduction that *hsf-1* RNAi causes a progeroid condition[7].

Next we examined whether effects of *daf-2(e1370)* on pathology are *daf-16* dependent, and found that they are, fully (Fig. 4f-j). This raises the question of where, within *daf-2(e1370)* mutants, *daf-16(+)* acts to prevent pathology development. With respect to lifespan, effects of *daf-16* are exerted most strongly in the intestine, as shown using transgenic strains where *daf-16* is expressed from tissue-specific promoters in a *daf-16; daf-2* background[20]. We used the same strains to ask: is this also true of the action of *daf-16* against senescent pathologies?

Expressing *daf-16* with its own promoter restored the capacity of *daf-2(e1370)* to suppress pathology in all four cases, confirming the anti-pathogenic role of *daf-16* (Supplementary Fig. 5a-d). In most cases, *daf-16* expression in muscle or in the nervous system had little effect on any pathologies (Supplementary Fig. 5a-d). By contrast, intestinal rescue of *daf-16* partially restored *daf-2* suppression of intestinal atrophy and yolk steatosis, suggesting an organ autonomous effect of *daf-16*. Intestine-limited rescue of *daf-16* did not suppress gonadal or pharyngeal pathology, suggesting that *daf-16* acts in a tissue- or organ-autonomous manner to suppress pathology.

That *daf-16* rescue in the intestine suppressed both intestinal atrophy and PLP formation (Supplementary Fig. 5d,e) is consistent with tissue autonomous suppression of conversion of intestinal biomass into yolk by *daf-16* (Ref 11). By contrast, intestinal atrophy is also modestly suppressed by neuronal rescue of *daf-16* (Supplementary Fig. 5d,e), implying tissue non-autonomous action of *daf-16*. Notably, neuronal (as well as intestinal) knockdown of DAF-2 auxin-induced DAF-2 degradation increases lifespan[21–23] while neuronal rescue of *daf-2* shortens it[24]; possibly intestinal *daf-16* effects on gut-to-yolk biomass conversion play a role in this.

Effects on senescent pathology of other interventions that extend lifespan

Next we investigated effects on senescent pathology of life-extending genetic interventions that inhibit protein synthesis, the mTOR pathway and mitochondrial function. Knockdown

of genes involved in protein translational machinery can increase lifespan, including the initiation factor 4F (eIF4F), *ife-2*, the translation initiators eIF2β (*iftb-1*) and eIF4G (*ifg-1*), and the small ribosomal subunit *rps-15* (Ref 25). We found that *ife-2* and *iftb-1* RNAi had little effect on pharyngeal pathology, gonad atrophy or uterine tumors. By contrast, both interventions suppressed intestinal atrophy, particularly *ife-2* RNAi (Supplementary Fig. 6a-e). Possibly this reflects reduction of vitellogenin synthesis, which is coupled to intestinal atrophy[11,14]. *iftb-1* RNAi also markedly increased PLP levels (Supplementary Fig. 6d) which was somewhat unexpected; however, inhibition of *iftb-1* can reduce brood size by 50% (Ref 26), which is predicted to cause yolk retention due to reduced efflux via egg laying[14].

Protein synthesis is promoted by the mechanistic target of rapamycin (mTOR) pathway, acting via ribosomal protein S6 kinase (S6K), encoded by *rsks-1* in *C. elegans*. Lifespan is increased by loss of function of several genes in the mTOR pathway, including *rsks-1(ok1255)* (Ref 27), which also reduces protein synthesis rate[28]. *rsks-1(ok1255)* slightly alleviated pathology development, with significant effects on pharyngeal decline and tumor formation. Intestinal atrophy appeared delayed but the effect was not statistically significant (Supplementary Fig. 7). The modest effects seen are consistent with the small magnitude of effects of *rsks-1* on lifespan, e.g. a 6% increase in mean lifespan in one study (20˚C, *E. coli* HT115)[28].

Finally, we tested mutations that perturb mitochondrial function and also delay development and increase lifespan, the Mit phenotype[29], specifically point mutations in *isp-1*, encoding a catalytic subunit of mitochondrial complex III (Ref 30) and *nuo-6*, a subunit of mitochondrial complex I (Ref 31). *isp-1(qm150)* and *nuo-6(qm200)* both inhibited pathology development of most but not all of the five pathologies (Supplementary Fig. 6f-j), indicating a major role of mitochondrial function in senescent pathogenesis.

One possibility is that mitochondrial metabolism supports processes that contribute to pathogenesis, such as feeding and reproduction. E.g., pharyngeal pumping contributes to pharyngeal pathology[32], production of oocytes past the reproductive period results in uterine tumors[33], and yolk synthesis results in PLP accumulation and intestinal atrophy[11]. Decreasing mitochondrial function reduces both pharyngeal pumping and reproduction[31,34,35].

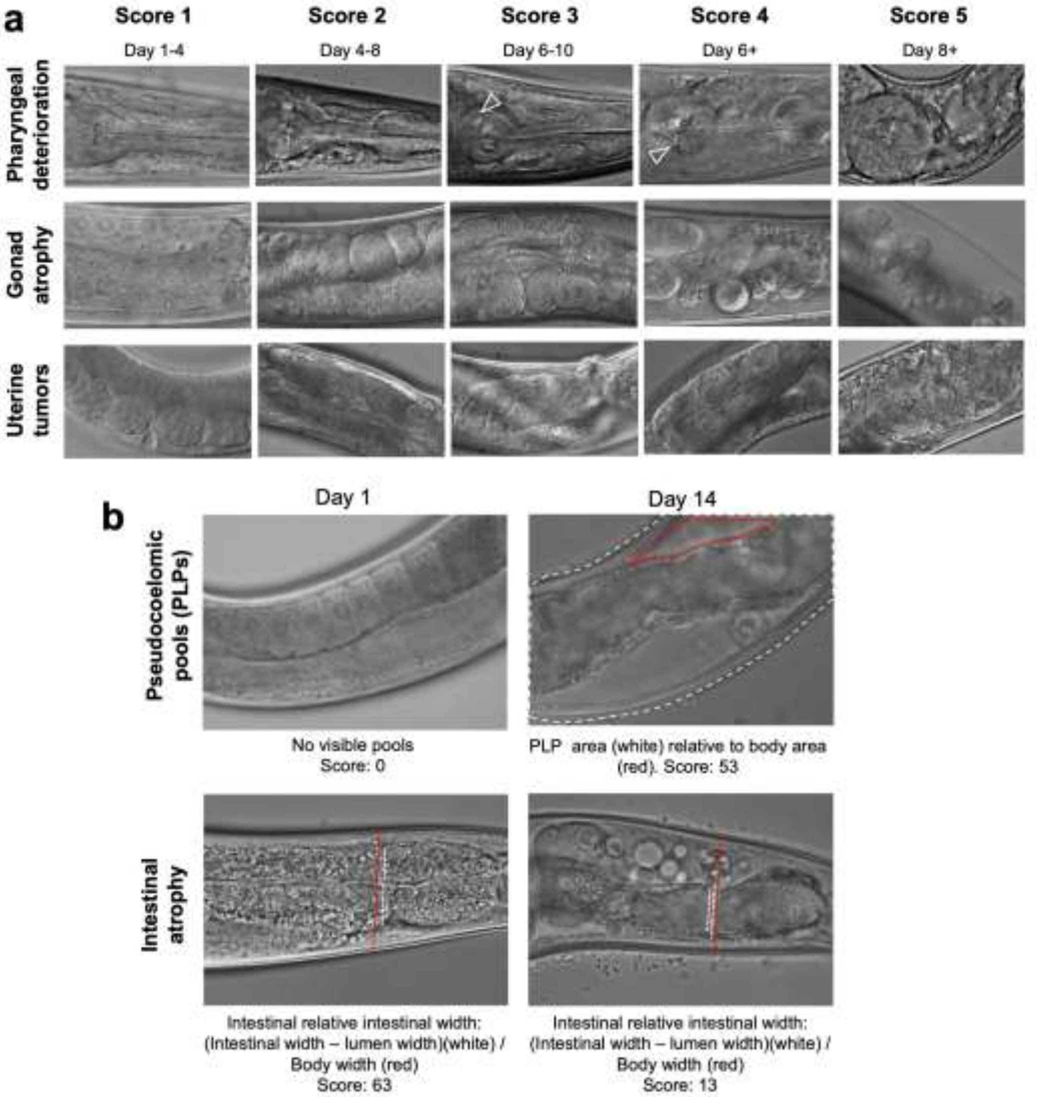

Supplementary Fig. 1 | Quantification of age-related pathologies in aging *C. elegans.* a Images showing age-related pathologies in *C. elegans* hermaphrodites: pharyngeal deterioration, gonad atrophy, uterine tumors, PLPs, and intestinal atrophy. Images were randomized, and given scores of 1-5. Here 1 = a youthful, healthy appearance; 2 = showing subtle signs of deterioration; 3 = clearly discernible, low level pathology; 4 = well developed pathology; and 5 = tissue so deteriorated as to be barely recognizable (e.g. gonad completely disintegrated), or reaching a maximal level (e.g. large tumor filling the entire body diameter). **b** Pseudocoelomic lipoprotein pool (PLP) formation (yolk accumulation) and intestinal atrophy. PLPs were measured by dividing the total area of yolk pools with the area of the body visible in the field of view. Intestinal atrophy was measured by calculating the width of intestinal tissue relative to body width.

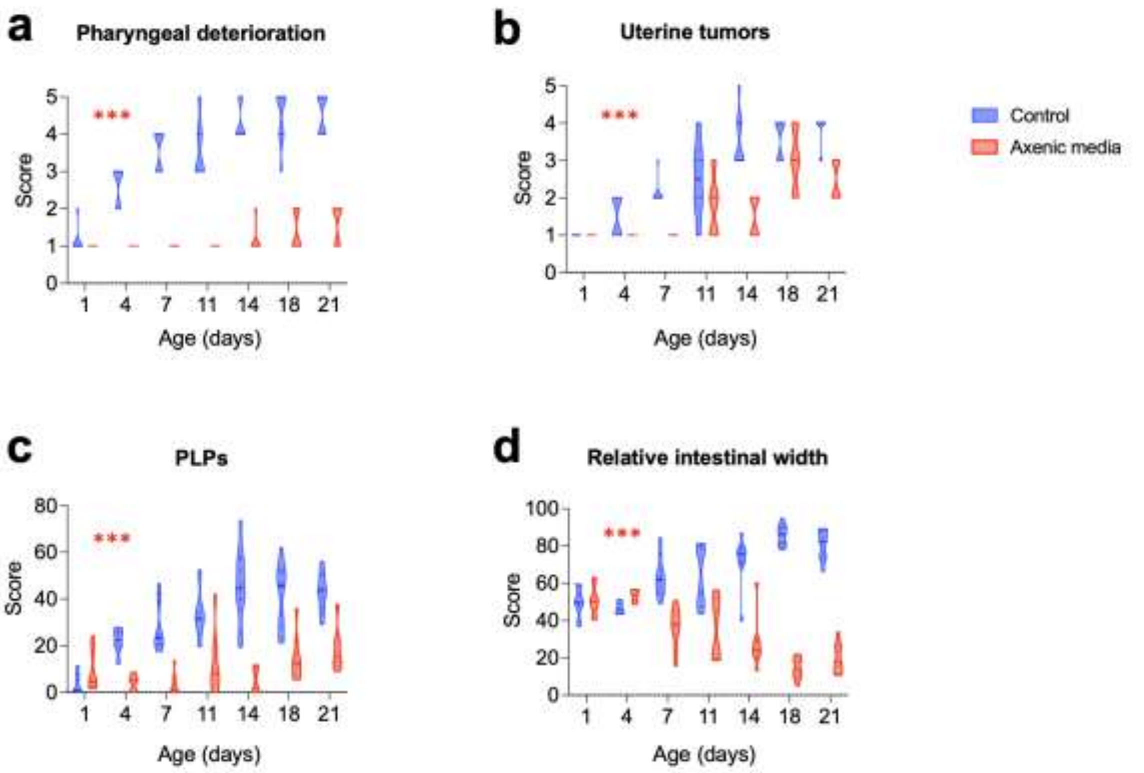

Supplementary Fig. 2 | Effects on senescent pathologies of axenic culture (putative DR regimen). a-d Culture on solid axenic medium suppresses all age-related pathologies. **a** Pharyngeal deterioration, **b** uterine tumors, **c** PLPs (yolk pools), **d** intestinal atrophy. Pooled data, N=2, n=10/trial. Stars show differences in pathology progression from day 1 to 14; two-way ANOVA (Bonferroni correction), and stars show differences in pathology progression via ANCOVA (Tukey correction). *** p<0.0001.

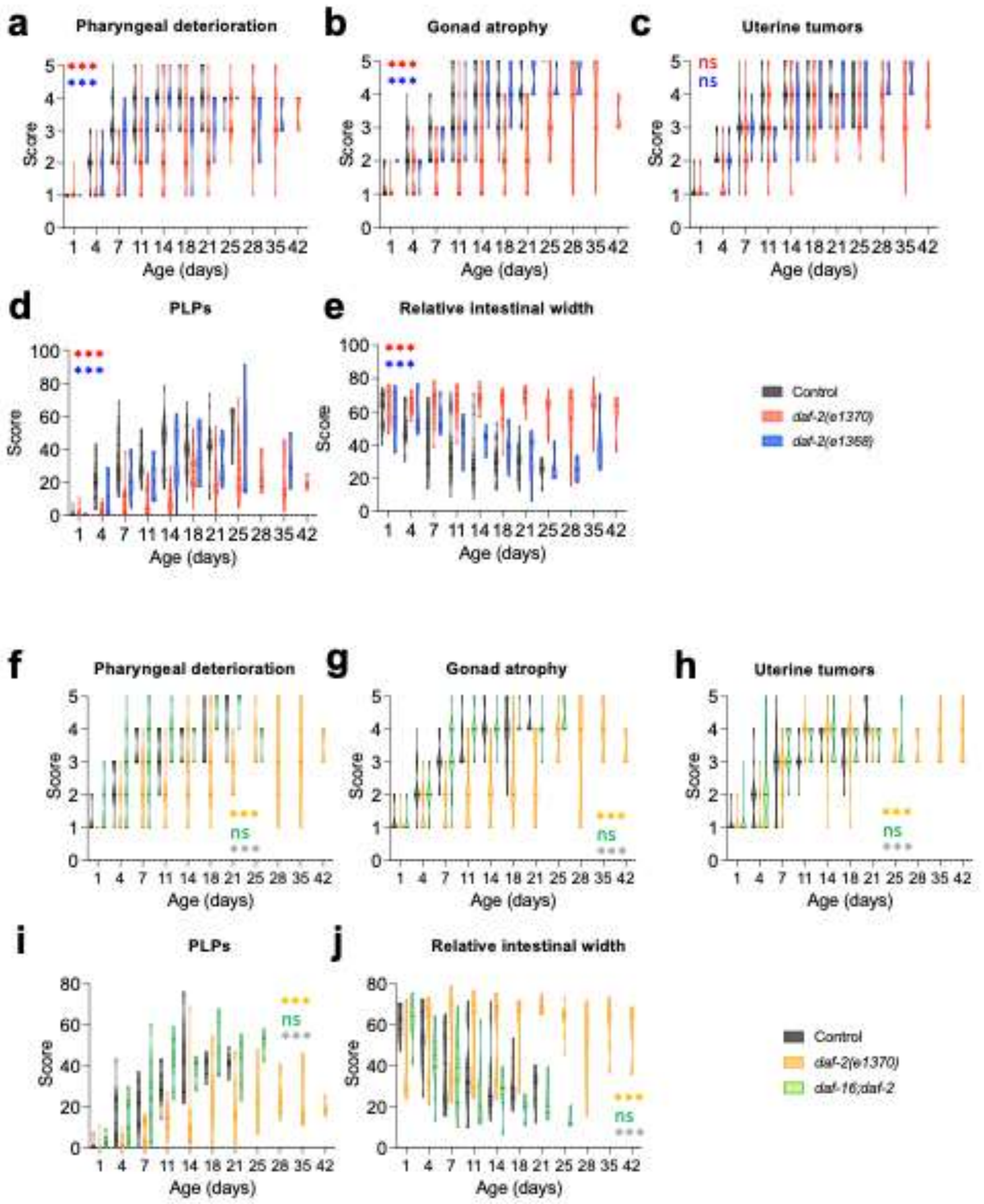

Supplementary Fig. 3 | Mutation of *daf-2* differentially suppresses senescent pathology. **a**-**e** *daf-2(e1370)* suppresses pathology development more strongly than *daf-2(e1368)*. **a** Pharyngeal deterioration, **b** gonad disintegration, **c** uterine tumors, **d** PLPs (yolk pools), **e** intestinal atrophy. **f**-**i** Pathology suppression by *daf-2(e1370)* is dependent on DAF-16. **f** Pharyngeal deterioration, **g** gonad disintegration, **h** uterine tumors, **i** PLPs, **j** gut atrophy. Pooled data, N=2, n=10/trial. Two-way ANOVA (Tukey correction), and stars denote differences in pathology progression via ANCOVA (Tukey correction). *** $p < 0.0001$. The color of the stars represents treatment color vs control. Grey stars, *daf-2* vs *daf-16; daf-2*.

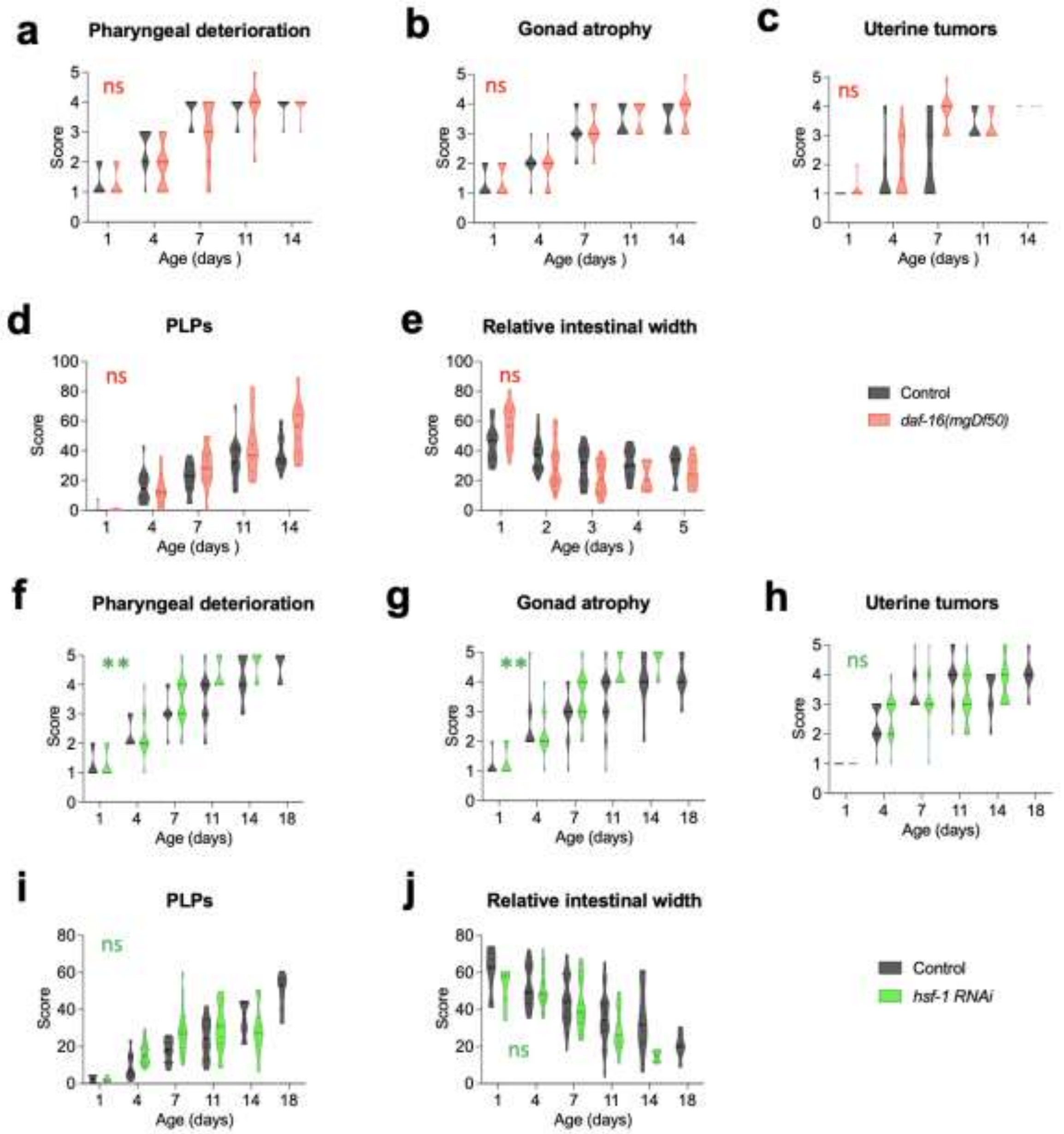

Supplementary Fig. 4 | Effects on pathology development of tissue-specific *daf-16* rescue and *hsf-1* inhibition. a-e *daf-16(mgDf50)* accelerates some but not all pathologies. **a** Pharyngeal deterioration, **b** gonad atrophy, **c** uterine tumors, **d** PLPs (yolk pools), **e** intestinal atrophy. **f-j** *hsf-1* RNAi accelerates some but not all pathologies. **f** Pharyngeal deterioration, **g** uterine tumors, **h** PLPs (yolk pools), **i** intestinal atrophy. Pooled data, N=2, n=10/trial. Two-way ANOVA (Bonferroni correction), and stars show differences in pathology progression via ANCOVA (Tukey correction). ** p<0.001.

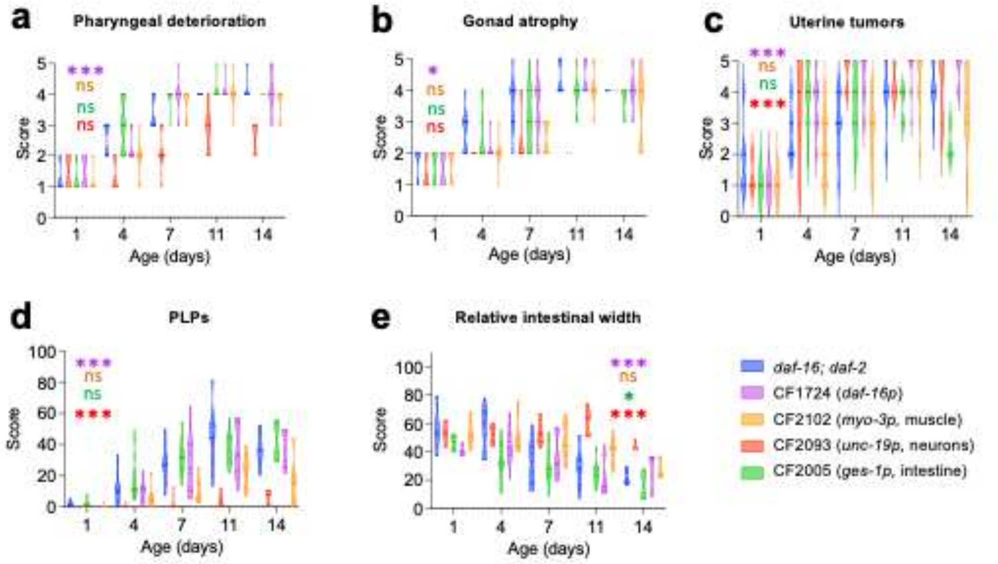

Supplementary Fig. 5 | Effects of tissue-specific rescue of *daf-16* on senescent pathology in *daf-16; daf-2(e1370)* mutants. *myo-3p::* muscle expressed; *ges-1p::* intestine expressed; and *unc-119p::* neuronally expressed. **a** Pharyngeal deterioration, **b** gonad atrophy, **c** uterine tumors, **d** PLPs (yolk pools), **e** intestinal atrophy. *N*=1, *n*=10. Two-way ANOVA (Tukey correction), and stars show differences in pathology progression via ANCOVA (Tukey correction), in comparison to the *daf-16; daf-2* control. The color of the stars represents treatment color vs control. * p<0.01; *** p<0.0001.

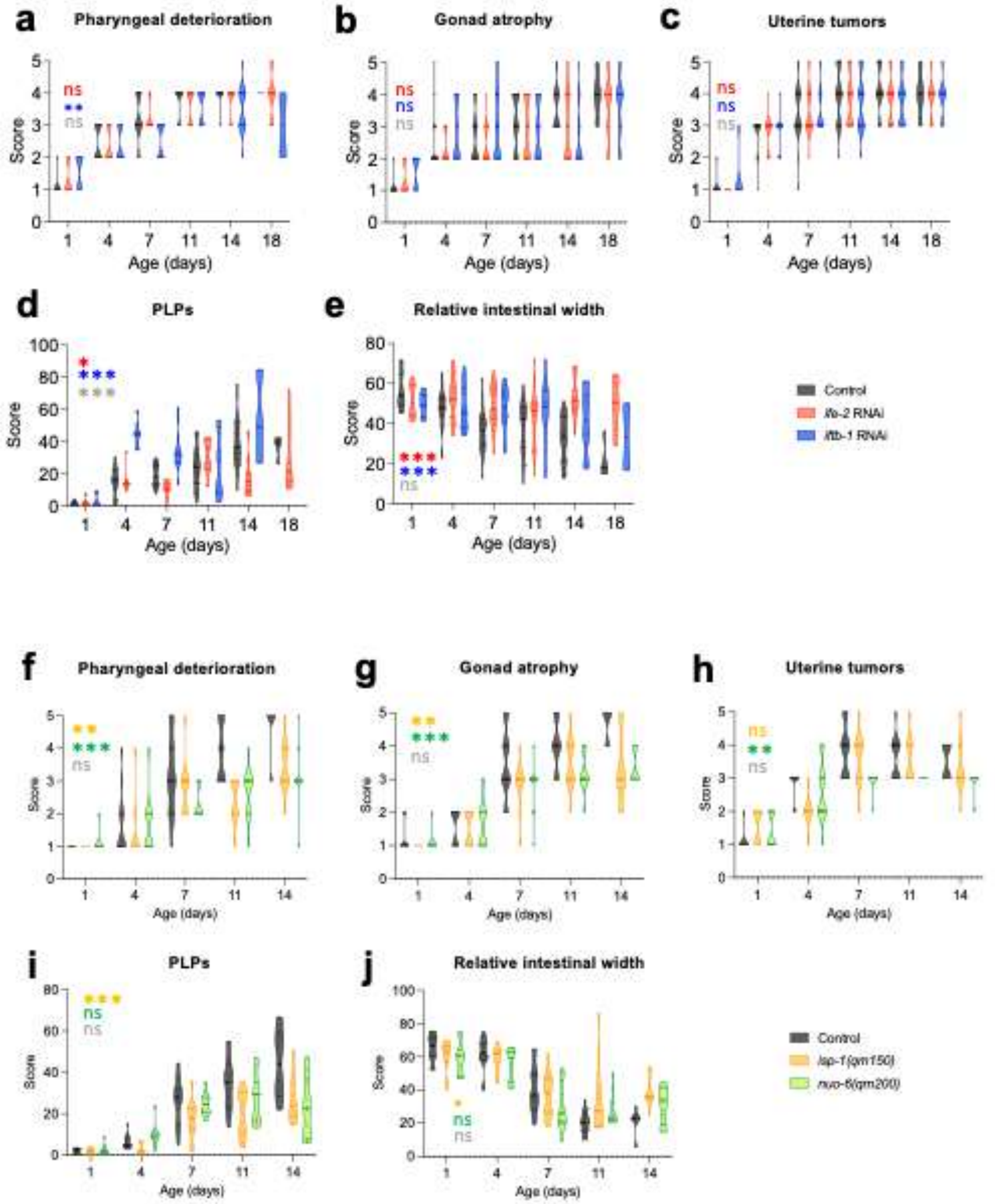

Supplementary Fig. 6. Evidence that protein synthesis and mitochondrial function promote pathology development. a-e *ife-2* and *iftb-1* RNAi inhibits gut atrophy but not most other pathologies. **a** Pharyngeal deterioration, **b** gonad disintegration, **c** uterine tumors, **d** PLPs (yolk pools), **e** intestinal atrophy. **f-j** *isp-1(qm150)* and *nuo-6(qm200)* inhibit multiple age-related pathologies. **f** Pharyngeal deterioration, **g** uterine tumors, **h** PLPs (yolk pools), **i** intestinal atrophy. Pooled data, N=2, n=10/trial. Two-way ANOVA (Tukey correction), and stars show differences in pathology progression via ANCOVA (Tukey correction). The color of the stars represents treatment color vs control. Grey stars: between treatment comparison. * $p < 0.01$; ** $p < 0.001$; *** $p < 0.0001$.

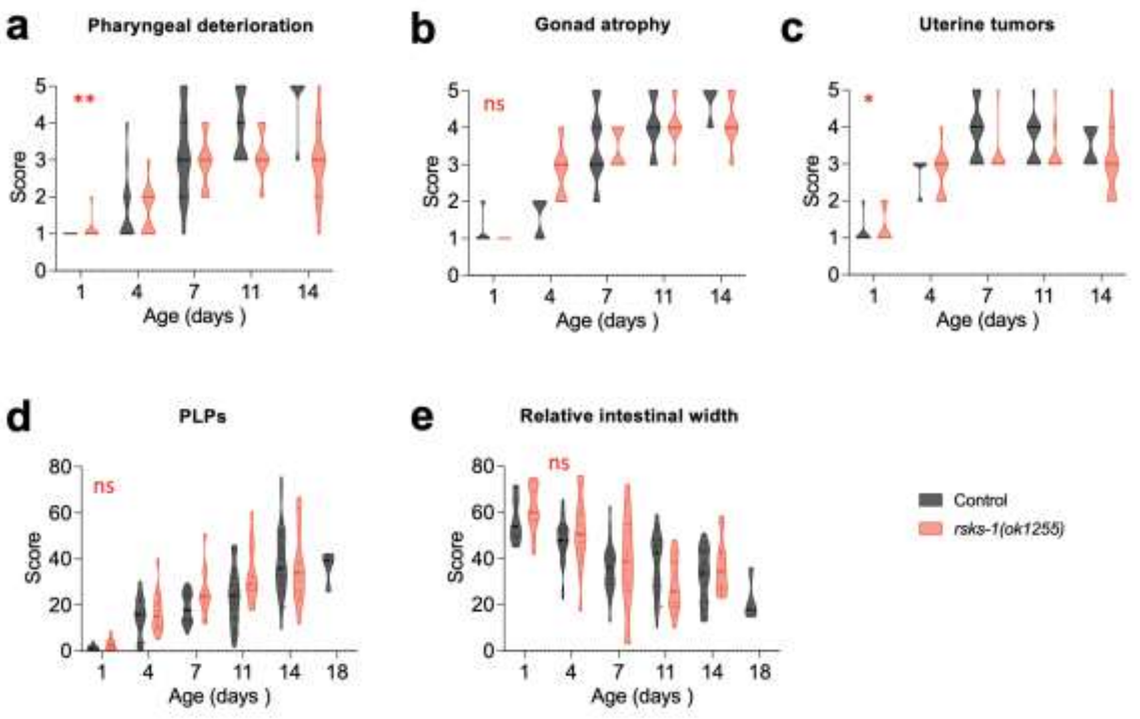

Supplementary Fig. 7 | Ribosomal protein S6 kinase has small effects on pathology development. a-e *rsks-1(ok1255)* suppresses pharyngeal decline and tumor formation but not other pathologies. **a** Pharyngeal deterioration, **b** gonad atrophy, **c** uterine tumors, **d** PLPs (yolk pools), **e** intestinal atrophy. Pooled data, N=2, n=10/trial. Two-way ANOVA (Tukey correction), and stars show differences in pathology progression via ANCOVA (Tukey correction). * p<0.01; ** p<0.001.

a

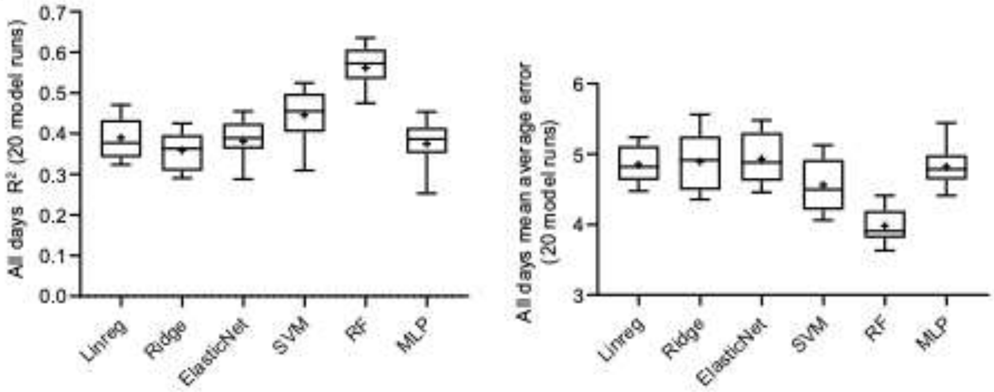

b

Feature	Coefficient
Day	1.403
Pharynx	-3.937
Gonad	-0.844
Tumor	-1.881
Yolk	0.431
Gut	1.253
Ind Term	12.534

c

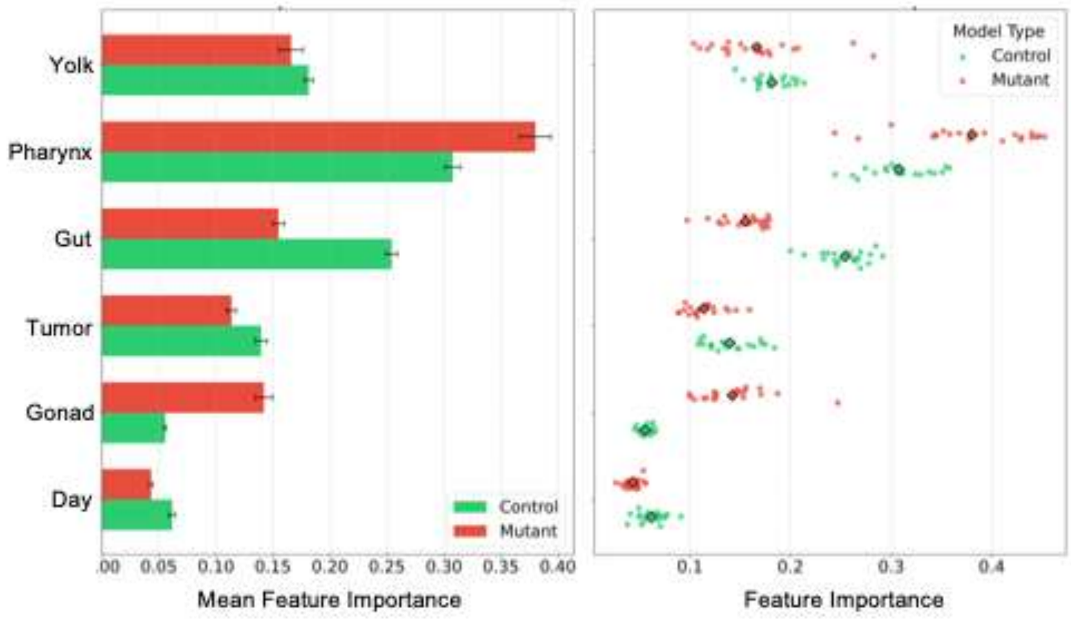

Supplementary Fig. 8 | Senescent pathology can be used to predict lifespan. a R^2 and mean average error (days) with different ML models, linear regression (Linreg), Ridge, ElasticNet regression, Support Vector Machine (SVM), Random Forest (RF), Multilayer Perceptron (MLP) independent of age at which the pathology was measured (i.e. pathology progression through time). **b** Coefficients generated by the linear regression model created by accounting for pathology progression to day 11 (c.f. Fig. 3a). **c** Feature importance: RF model Mean Decrease in Impurity (MDI); animals are split according to wild type (control) and mutant conditions (genetic mutations and RNAi). S.E.M. displayed following 20 model runs.

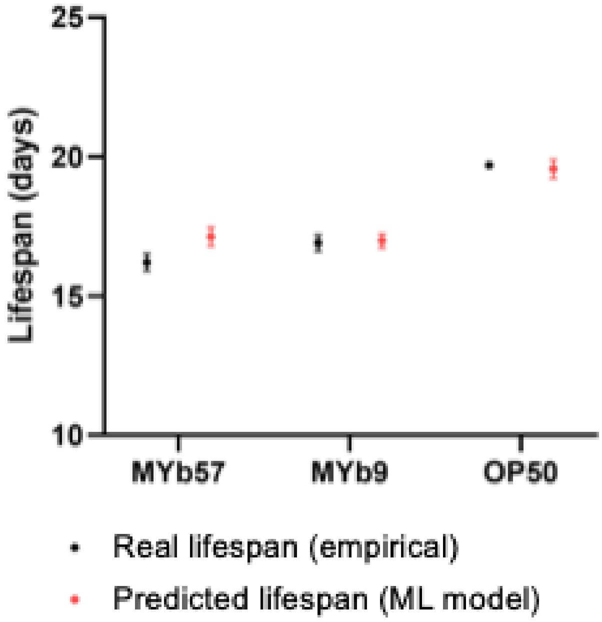

Supplementary Fig. 9. Predictive power of the ML model. Mean and SD of empirical lifespans, and lifespans predicted with the ML model, of animals cultivated with MYb57, MYb9 or *E. coli* OP50. Empirical observed: n=3 trials; ML predicted lifespan: n=20 model runs; multiple t-test. Predicted lifespans are found to be within 1 day of empirically observed lifespans. Error bars show standard deviation.

Score	Pharyngeal deterioration	Gonad atrophy	Uterine tumors
1	Healthy pharynx. Borders are smooth, radial muscle lines are visible and intact.	Healthy gonad. Gonad arms are intact. Edges are smooth. Germline cells are surrounded by cytoplasm. Gonad arms fill up the body cavity and are mostly touching each other.	Healthy uterus, no tumours.
2	Borders of the pharynx are uneven. Radial muscle lines are starting to fade. Small vacuoles/cavities appear.	Thinned gonad. Starting to show deterioration. Diameter of gonad arm is decreased and are touching less than in a healthy gonad. Edges of distal arm are starting to shrivel.	Abnormal uterus; possible beginning of tumor
3	Aged pharynx. One of the following aging features: bacterial plug present, terminal bulb starting to swell or cavities in bulb.	Aged gonad. Gonad even thinner. Further decrease of diameter and further shrivelling.	Small tumors; Area/(diameter)^2 < 0.9
4	Very aged. Two or more of the aging features above.	Very aged. Fragmented gonad. Disruption/displacement/ twisting of gonad	Large tumors but do not fill entire cavity; A/d^2 > 0.9
5	Posterior pharyngeal bulb completely swollen or disintegrated.	Gonad not recognisable.	Massive tumors; fills the entire diameter of body cavity

Supplementary Table 1. Pathology scoring system. C.f. Supplementary Fig. 1.

Treatment, species, genotype	Pathology data source	Lifespan data source
Individual N2 animals, maintained at 20˚C and followed throughout life	This study	This study
N2 maintained at 15˚C	This study	(Miller et al., 2017)
N2 maintained at 25˚C	This study	(Miller et al., 2017)
N2 maintained on solid axenic medium	This study	(Lenaerts et al., 2008)
Mated N2 hermaphrodites	This study	(Gems and Riddle, 1996)
N2 males	This study	(Gems and Riddle, 2000)
N2 hermaphrodites	(Kern et al., 2023)	(Kern et al., 2023)
C. inopinata females	(Kern et al., 2023)	(Kern et al., 2023)
C. briggsae hermaphrodites	(Kern et al., 2023)	(Kern et al., 2023)
C. nigoni females	(Kern et al., 2023)	(Kern et al., 2023)
C. tropicalis hermaphrodites	(Kern et al., 2023)	(Kern et al., 2023)
C. wallacei females	(Kern et al., 2023)	(Kern et al., 2023)
C. remanei females	This study	Unpublished
Pristionchus pacificus hermaphrodites	(Kern et al., 2023)	(Kern et al., 2023)
Pristionchus exspectatus females	(Kern et al., 2023)	(Kern et al., 2023)
Laser ablation z1-4 *C. elegans*	(Kern et al., 2023)	(Kern et al., 2023)
Laser ablation z2,3 *C. briggsae*	(Kern et al., 2023)	(Kern et al., 2023)
Laser ablation z2,3 *C. elegans*	(Kern et al., 2023)	(Kern et al., 2023)
Laser ablation z2,3 *C. tropicalis*	(Kern et al., 2023)	(Kern et al., 2023)
Laser ablation z2,3 *P. pacificus*	(Kern et al., 2023)	(Kern et al., 2023)
glp-1(bn4)	(Kern et al., 2023)	(Zhao et al., 2017)
glp-4(bn2)	(Kern et al., 2023)	(Arantes-Oliveira et al., 2002)
daf-2(e1370)	This study	(Bansal et al., 2014)
daf-2(e1368)	This study	(Ezcurra et al., 2018)
daf-16(mgDf50); daf-2(e1370)	This study	(Bansal et al., 2014)
daf-16(mgDf50)	This study	(Bansal et al., 2014)
Pdaf-16::daf-16; daf-16(mu86)	This study	(Libina et al., 2003)
Pges-1::daf-16; daf-16(mu86)	This study	(Libina et al., 2003)
Pmyo-3::daf-16; daf-16(mu86)	This study	(Libina et al., 2003)
Punc-119::daf-16; daf-16(mu86)	This study	(Libina et al., 2003)
mab-3(mu15)	(Ezcurra et al., 2018)	(Ezcurra et al., 2018)
mab-3(mu15) males	(Ezcurra et al., 2018)	(Ezcurra et al., 2018)
him-5(e1490)	This study	(Ezcurra et al., 2018)
him-5(e1490) males	This study	(Ezcurra et al., 2018)
fem-3(e2006)	(Kern et al., 2023)	(Kern et al., 2023)
fog-2(q71)	(Kern et al., 2023)	(Kern et al., 2023)
rps-15 RNAi	This study	(Hansen et al., 2007)
iftb-1 RNAi	This study	(Hansen et al., 2007)
rsks-1(ok1255)	This study	(Pan et al., 2007)
isp-1(qm150)	This study	(Yang and Hekimi, 2010)
nuo-6(qm200)	This study	(Yang and Hekimi, 2010)
hsf-1 RNAi	This study	(Morley and Morimoto, 2004)
ife-2 RNAi	This study	(Syntichaki et al., 2007)
iftb-1 RNAi	This study	(Hansen et al., 2007)
vit-5 RNAi	(Sornda et al., 2019)	(Sornda et al., 2019)
vit-6 RNAi	(Sornda et al., 2019)	(Sornda et al., 2019)
vit-5, vit-6 RNAi	(Sornda et al., 2019)	(Sornda et al., 2019)

Supplementary Table 2. All lifespan data sources and pathology data sources collated and used in ML analysis. C.f. Fig. 3.

Supplementary references

1. Fontana, L. & Partridge, L. Promoting health and longevity through diet: From model organisms to humans. *Cell* **161**, 106–118 (2015).
2. Kapahi, P., Kaeberlein, M. & Hansen, M. Dietary restriction and lifespan: Lessons from invertebrate models. *Ageing Res. Rev.* **39**, 3–14 (2017).
3. Vanfleteren, J. R. & Braeckman, B. P. Mechanisms of life span determination in *Caenorhabditis elegans*. *Neurobiol. Aging* **20**, 487–502 (1999).
4. Walker, G., Houthoofd, K., Vanfleteren, J. R. & Gems, D. Dietary restriction in *C. elegans*: From rate-of-living effects to nutrient sensing pathways. *Mech. Ageing Dev.* **126**, 929–937 (2005).
5. Lenaerts, I., Walker, G. A., Van Hoorebeke, L., Gems, D. & Vanfleteren, J. R. Dietary restriction of *Caenorhabditis elegan*s by axenic culture reflects nutritional requirement for constituents provided by metabolically active microbes. *J. Gerontol. A Biol. Sci. Med. Sci.* **63**, 242–252 (2008).
6. Kenyon, C. J. The genetics of ageing. *Nature* **464**, 504–512 (2010).
7. Garigan, D. *et al.* Genetic analysis of tissue aging in *Caenorhabditis elegans*: A role for heat-shock factor and bacterial proliferation. *Genetics* **161**, 1101–1112 (2002).
8. Podshivalova, K., Kerr, R. A. & Kenyon, C. How a mutation that slows aging can also disproportionately extend end-of-life decrepitude. *Cell Rep.* **19**, 441–450 (2017).
9. Zhao, Y. *et al.* Mutation of *daf - 2* extends lifespan via tissue-specific effectors that suppress distinct life-limiting pathologies. *Aging Cell* **20**, 15458 (2021).
10. DePina, A. S. *et al.* Regulation of *Caenorhabditis elegans* vitellogenesis by DAF-2/IIS through separable transcriptional and posttranscriptional mechanisms. *BMC Physiol.* **11**, 11 (2011).
11. Ezcurra, M. *et al. C. elegans* eats its own intestine to make yolk leading to multiple senescent pathologies. *Curr. Biol.* **28**, 2544-2556 (2018).
12. Gems, D. & Riddle, D. L. Genetic, behavioral and environmental determinants of male longevity in *Caenorhabditis elegans*. *Genetics* **154**, 1597–1610 (2000).
13. Murphy, C. T. *et al.* Genes that act downstream of DAF-16 to influence the lifespan of *Caenorhabditis elegans*. *Nature* **424**, 277–283 (2003).
14. Sornda, T. *et al.* Production of YP170 vitellogenins promotes intestinal senescence in *C. elegans*. *J. Gerontol. A Biol. Sci. Med. Sci.* **74**, 1180-1188 (2019) .
15. Gems, D. *et al.* Two pleiotropic classes of *daf-2* mutation affect larval arrest, adult behavior, reproduction and longevity in *Caenorhabditis elegans*. *Genetics* **150**, 129–155 (1998).
16. Golden, T. R. *et al.* Dramatic age-related changes in nuclear and genome copy number in the nematode *Caenorhabditis elegans*. *Aging Cell* **6**, 179–188 (2007).
17. Kenyon, C., Chang, J. & Gensch, E. A *C. elegan*s mutant that lives twice as long as wild type. *Nature* **366**, 461–464 (1993).
18. Lin, K., Hsin, H., Libina, N. & Kenyon, C. Regulation of the *Caenorhabditis elegans* longevity protein DAF-16 by insulin/IGF-1 and germline signaling. *Nat. Genet.* **28**, 139–145 (2001).

19. Hsu, A.-L., Murphy, C. T. & Kenyon, C. Regulation of aging and age-related disease by DAF-16 and heat-shock factor. *Science* **300**, 1142–1145 (2003).
20. Libina, N., Berman, J. R. & Kenyon, C. Tissue-specific activities of *C. elegans* DAF-16 in the regulation of lifespan. *Cell* **115**, 489–502 (2003).
21. Roy, C. *et al.* DAF-2/insulin IGF-1 receptor regulates motility during aging by integrating opposite signaling from muscle and neuronal tissues. *Aging Cell* **21**, e13660 (2022).
22. Venz, R., Pekec, T., Katic, I., Ciosk, R. & Ewald, C. Y. End-of-life targeted degradation of DAF-2 insulin/IGF-1 receptor promotes longevity free from growth-related pathologies. *eLife* **10**, e71335 (2021).
23. Zhang, Y.-P. *et al.* Intestine-specific removal of DAF-2 nearly doubles lifespan in *Caenorhabditis elegans* with little fitness cost. *Nat. Commun.* **13**, 6339 (2022).
24. Wolkow, C. A., Kimura, K. D., Lee, M.-S. & Ruvkun, G. Regulation of *C. elegans* life-span by insulinlike signaling in the nervous system. *Science* **290**, 147–150 (2000).
25. Hansen, M. *et al.* Lifespan extension by conditions that inhibit translation in *Caenorhabditis elegans*. *Aging Cell* **6**, 95–110 (2007).
26. Zhang, Y. & Maduzia, L. L. Mutations in *Caenorhabditis elegans* eIF2β permit translation initiation from non-AUG start codons. *Genetics* **185**, 141–152 (2010).
27. Syntichaki, P., Troulinaki, K. & Tavernarakis, N. eIF4E function in somatic cells modulates ageing in *Caenorhabditis elegans*. *Nature* **445**, 922–926 (2007).
28. Pan, K. Z. *et al.* Inhibition of mRNA translation extends lifespan in *Caenorhabditis elegans*. *Aging Cell* **6**, 111–119 (2007).
29. Rea, S. L. Metabolism in the *Caenorhabditis elegans* Mit mutants. *Exp. Gerontol.* **40**, 841–849 (2005).
30. Feng, J., Bussière, F. & Hekimi, S. Mitochondrial electron transport is a key determinant of life span in *Caenorhabditis elegans*. *Dev. Cell.* **1**, 633–644 (2001).
31. Yang, W. & Hekimi, S. Two modes of mitochondrial dysfunction lead independently to lifespan extension in *Caenorhabditis elegans*. *Aging Cell* **9**, 433–447 (2010).
32. Zhao, Y. *et al.* Two forms of death in ageing *Caenorhabditis elegans*. *Nat. Commun.* **8**, 15458 (2017).
33. Wang, H. *et al.* A parthenogenetic quasi-program causes teratoma-like tumors during aging in wild-type *C. elegans*. *NPJ Aging Mech. Dis.* **4**, 6 (2018).
34. Jafari, G. *et al.* Tether mutations that restore function and suppress pleiotropic phenotypes of the *C. elegans isp-1(qm150)* Rieske iron–sulfur protein. *Proc. Natl. Acad. Sci. U S A* **112**, E6148-E6157 (2015).
35. Yee, C., Yang, W. & Hekimi, S. The intrinsic apoptosis pathway mediates the pro-longevity response to mitochondrial ROS in *C. elegans*. *Cell* **157**, 897–909 (2014).
