# Supplementary material for: Machine learning predicts lifespan and suggests underlying causes of death in aging C. elegans
Source: Commun Biol. 2025 Nov 21;8:1630. doi: 10.1038/s42003-025-09012-9 (PMC12638908; doi:10.1038/s42003-025-09012-9)
Supplement: Supplementary file 3 — Description of Additional Supplementary Files [file 42003_2025_9012_MOESM3_ESM.pdf]

Description of Additional Supplementary files

File name: Supplementary Data 1

Description: Raw pathology and lifespan data original to this study.

File name: Supplementary Data 2

Description: Raw data used to build the ML model.
