# Supplementary material for: Machine learning predicts lifespan and suggests underlying causes of death in aging C. elegans
Source: Commun Biol. 2025 Nov 21;8:1630. doi: 10.1038/s42003-025-09012-9 (PMC12638908; doi:10.1038/s42003-025-09012-9)
Supplement: Supplementary file 6 — Reporting Summary [file 42003_2025_9012_MOESM6_ESM.pdf]

nature portfolio

Corresponding author(s): Marina Ezcurra and David Gems

Last updated by author(s): 25/9/2025

Reporting Summary

Nature Portfolio wishes to improve the reproducibility of the work that we publish. This form provides structure for consistency and transparency in reporting. For further information on Nature Portfolio policies, see our Editorial Policies and the Editorial Policy Checklist.

Statistics

For all statistical analyses, confirm that the following items are present in the figure legend, table legend, main text, or Methods section.

n/a	Confirmed	
☐	☒	The exact sample size (*n*) for each experimental group/condition, given as a discrete number and unit of measurement
☐	☒	A statement on whether measurements were taken from distinct samples or whether the same sample was measured repeatedly
☐	☒	The statistical test(s) used AND whether they are one- or two-sided<br>*Only common tests should be described solely by name; describe more complex techniques in the Methods section.*
☒	☐	A description of all covariates tested
☐	☒	A description of any assumptions or corrections, such as tests of normality and adjustment for multiple comparisons
☐	☒	A full description of the statistical parameters including central tendency (e.g. means) or other basic estimates (e.g. regression coefficient) AND variation (e.g. standard deviation) or associated estimates of uncertainty (e.g. confidence intervals)
☐	☒	For null hypothesis testing, the test statistic (e.g. *F*, *t*, *r*) with confidence intervals, effect sizes, degrees of freedom and *P* value noted<br>*Give P values as exact values whenever suitable.*
☒	☐	For Bayesian analysis, information on the choice of priors and Markov chain Monte Carlo settings
☒	☐	For hierarchical and complex designs, identification of the appropriate level for tests and full reporting of outcomes
☐	☒	Estimates of effect sizes (e.g. Cohen's *d*, Pearson's *r*), indicating how they were calculated

Our web collection on statistics for biologists contains articles on many of the points above.

Software and code

Policy information about availability of computer code

Data collection	Image acquisition driven using Volocity 6.3 software
Data analysis	ImageJ was used for image analysis. Custom made code used for data analysis and is provided.

For manuscripts utilizing custom algorithms or software that are central to the research but not yet described in published literature, software must be made available to editors and reviewers. We strongly encourage code deposition in a community repository (e.g. GitHub). See the Nature Portfolio guidelines for submitting code & software for further information.

Data

Policy information about availability of data

All manuscripts must include a data availability statement. This statement should provide the following information, where applicable:

- Accession codes, unique identifiers, or web links for publicly available datasets
- A description of any restrictions on data availability
- For clinical datasets or third party data, please ensure that the statement adheres to our policy

Raw data used to generate ML model available in Supplementary datasets. Link provided to the code used to generate ML model.

Research involving human participants, their data, or biological material

Policy information about studies with human participants or human data. See also policy information about sex, gender (identity/presentation), and sexual orientation and race, ethnicity and racism.

Reporting on sex and gender	NA
Reporting on race, ethnicity, or other socially relevant groupings	NA
Population characteristics	NA
Recruitment	NA
Ethics oversight	NA

Note that full information on the approval of the study protocol must also be provided in the manuscript.

Field-specific reporting

Please select the one below that is the best fit for your research. If you are not sure, read the appropriate sections before making your selection.

☒ Life sciences ☐ Behavioural & social sciences ☐ Ecological, evolutionary & environmental sciences

For a reference copy of the document with all sections, see nature.com/documents/nr-reporting-summary-flat.pdf

Life sciences study design

All studies must disclose on these points even when the disclosure is negative.

Sample size	Sample sizes were determined based on commonly used standards in C. elegans ageing and physiology research, and reflect the minimum number of biological replicates required to achieve statistical power for detecting biologically meaningful differences. No formal a priori power calculation was performed.
Data exclusions	Criteria were established a priori: Animals not imaged if they experienced internal hatching or intestinal rupture.
Replication	Each experiment was conducted in duplicate or triplicate to ensure replication. Values from the individual trials are reported.
Randomization	Conditions were imaged in an alternating order across replicates to control for potential bias related to imaging sequence.
Blinding	Scoring of images was performed blinded.

Reporting for specific materials, systems and methods

We require information from authors about some types of materials, experimental systems and methods used in many studies. Here, indicate whether each material, system or method listed is relevant to your study. If you are not sure if a list item applies to your research, read the appropriate section before selecting a response.

Materials & experimental systems

n/a	Involved in the study	
☒	☐	Antibodies
☒	☐	Eukaryotic cell lines
☒	☐	Palaeontology and archaeology
☐	☒	Animals and other organisms
☒	☐	Clinical data
☒	☐	Dual use research of concern
☒	☐	Plants

Methods

n/a	Involved in the study	
☒	☐	ChIP-seq
☒	☐	Flow cytometry
☒	☐	MRI-based neuroimaging

Animals and other research organisms

Policy information about studies involving animals; ARRIVE guidelines recommended for reporting animal research, and Sex and Gender in Research

Laboratory animals	C. elegans strains used in this study included: N2 hermaphrodite stock, N2M male stock fln-2(ot611) X (Ref. 47), CB3844 fem-3(e2006) IV; CF1724 daf-16(mu86) I; daf-2(e1370) III; muIs105 [daf-16p::GFP::daf-16 + rol-6(su1006)], CF2005 daf-16(mu86) I; daf-2(e1370) III; muIs120 [ges-1p::GFP::daf-16 + rol-6(su1006)], CF2093 daf-16(mu86) I; daf-2(e1370) III; muIs131 [unc-119p::GFP::daf-16 + rol-6(su1006)], CF2102 daf-16(mu86) I; daf-2(e1370) III; muIs126 [myo-3p::GFP::daf-16 + rol-6(su1006)], DR466 him-5(e1490) V, DR1563 daf-2(e1370) III, DR1572 daf-2(e1368) III, GA14 fog-2(q71) V; GA114 daf-16(mgDf50) I; daf-2(e1370) III, GR1307 daf-16(mgDf50) I, MQ887 isp-1(qm15) IV, MQ1333 nuo-6(qm200) I and RB1206 rsks-1(ok1255) III.
Wild animals	The study did not involve wild animals
Reporting on sex	C. elegans hermaphrodites were mostly used in the study, this is standard procedure in the field. We compared hermaphrodites and males in one set of experiments.
Field-collected samples	The study did not involve field-collected samples.
Ethics oversight	Ethical approval was not required for this study as Caenorhabditis elegans is an invertebrate species not covered under the UK Animals (Scientific Procedures) Act 1986. Tthe use of C. elegans in research does not require regulation or formal ethical approval in the United Kingdom.

Note that full information on the approval of the study protocol must also be provided in the manuscript.

Plants

Seed stocks	NA
Novel plant genotypes	NA
Authentication	NA
